# A self-training program for sensory substitution devices

**DOI:** 10.1371/journal.pone.0250281

**Published:** 2021-04-27

**Authors:** Galit Buchs, Benedetta Haimler, Menachem Kerem, Shachar Maidenbaum, Liraz Braun, Amir Amedi

**Affiliations:** 1 The Baruch Ivcher Institute For Brain, Cognition & Technology, The Baruch Ivcher School of Psychology, Interdisciplinary Center (IDC), Herzeliya, Israel; 2 Department of Cognitive Science, Faculty of Humanities, Hebrew University of Jerusalem, Jerusalem, Israel; 3 Center of Advanced Technologies in Rehabilitation (CATR), The Chaim Sheba Medical Center, Ramat Gan, Israel; 4 Department of Biomedical Engineering, Ben Gurion University, Beersheba, Israel; 5 Hebrew University of Jerusalem, Jerusalem, Israel; Midwestern University, UNITED STATES

## Abstract

Sensory Substitution Devices (SSDs) convey visual information through audition or touch, targeting blind and visually impaired individuals. One bottleneck towards adopting SSDs in everyday life by blind users, is the constant dependency on sighted instructors throughout the learning process. Here, we present a proof-of-concept for the efficacy of an online self-training program developed for learning the basics of the EyeMusic visual-to-auditory SSD tested on sighted blindfolded participants. Additionally, aiming to identify the best training strategy to be later re-adapted for the blind, we compared multisensory vs. unisensory as well as perceptual vs. descriptive feedback approaches. To these aims, sighted participants performed identical SSD-stimuli identification tests before and after ~75 minutes of self-training on the EyeMusic algorithm. Participants were divided into five groups, differing by the feedback delivered during training: auditory-descriptive, audio-visual textual description, audio-visual perceptual simultaneous and interleaved, and a control group which had no training. At baseline, before any EyeMusic training, participants SSD objects’ identification was significantly above chance, highlighting the algorithm’s intuitiveness. Furthermore, self-training led to a significant improvement in accuracy between pre- and post-training tests in each of the four feedback groups versus control, though no significant difference emerged among those groups. Nonetheless, significant correlations between individual post-training success rates and various learning measures acquired during training, suggest a trend for an advantage of multisensory vs. unisensory feedback strategies, while no trend emerged for perceptual vs. descriptive strategies. The success at baseline strengthens the conclusion that cross-modal correspondences facilitate learning, given SSD algorithms are based on such correspondences. Additionally, and crucially, the results highlight the feasibility of self-training for the first stages of SSD learning, and suggest that for these initial stages, unisensory training, easily implemented also for blind and visually impaired individuals, may suffice. Together, these findings will potentially boost the use of SSDs for rehabilitation.

## Introduction

Finding ways to convey visual information to the millions of blind individuals worldwide is a major rehabilitation goal [1]. There are many efforts in this direction [2]. One promising set of tools in this domain are visual-to-auditory Sensory Substitution Devices (SSDs). Visual-to-auditory SSDs are a family of non-invasive devices that convert visual input to audition according to a specific algorithm [3, 4]. SSDs have already shown their potential to aid blind individuals in various scenarios. For example, blind SSD users successfully performed navigation tasks [5–7], obstacle detection and avoidance [8] as well as various object recognition tasks with different degrees of difficulty while using SSDs [9]. Most of the studies with SSDs were conceived for research purposes, thus limiting the use of these devices to lab settings, even though there are some examples of SSD super users who managed to successfully use SSDs also in real life [10]. However, despite all these promising outcomes, SSDs have not been widely adopted by the blind and visually impaired communities [9, 11]. What has prevented their adoption?

Some previously suggested reasons included the lack of availability, cost and cumbersomeness of the setups [11, 12]. However, these issues have been mitigated to a large extent by the rise in availability of smartphones enabling mobile compact and relatively cheap processing and sensing units. Visual-to-auditory SSDs such as the vOICe [4] are freely available and do not require additional hardware beyond regular headphones. The main issue currently highlighted as the bottleneck to SSDs wide adoption is the training necessary in order to master them [9, 13]. Indeed, SSDs algorithms are generally quite complex to interpret, especially for understanding finer grained differentiations and cluttered images, thus constantly requiring the presence of a sighted instructor who will teach the trainee (blind/ sighted/ the researchers themselves) how to interpret the SSD information and understand the visual information that is presented to them in both advanced and basic training. Specifically, the dependency on a sighted instructor obviously applies to advanced SSD training programs which one can imagine might require more instructions and explanations by the instructors, e.g., to explain how visual concepts such as depth are transformed by the SSD algorithm, especially to congenitally blind users who might not be familiar with such concepts at all, thus creating intensive training programs [14]. However, due to the lack of alternative available training approaches, sighted instructors are constantly required also during beginners’ programs, namely, during the initial stages of SSD learning including the learning of the main features of the SSD transformation algorithm, interpreting simple shapes, learning to interpret spatial cues conveyed by the SSD, etc. Note that this basic training is still required despite SSD main features being based on cross-modal correspondences, which potentially allow a certain degree of intuitive learning in the users making this stage faster and easier [3, 15, 16]. Both of these types of training require automation and standardization, but pose different challenges. As the transformation aspect is relevant for all potential users, sighted, late blind, congenital blind, and individuals with residual vision, we here focus on the second type–the basic training on the transformation.

To explore possible ways to reduce the training challenge, we present here the results of a proof-of-concept study, where we tested on sighted blindfolded participants, the feasibility of learning the basic principles of the EyeMusic, a visual-to-auditory-SSD developed in our lab [3], through a self-training, free and accessible program we developed. In addition, we also aimed at identifying the most effective feedback strategy to be deployed during training, namely a strategy maximizing the outcome of such self-learning. To these aims, our study included five different groups of sighted participants, four training groups and one control group. All training groups undertook identical pre- and post-training auditory tests on stimuli identification conveyed via the EyeMusic SSD alone, with ~75 minutes of self-training on the basic features of this SSD in between these tests. During training, participants were exposed to different feedback strategies: one group was exposed to auditory feedback, forming a unisensory training group (hearing an EyeMusic stimulus and receiving auditory descriptions of what was just heard) and three groups were exposed to visual feedback, forming multisensory training groups (hearing an EyeMusic stimulus and receiving three different forms of visual feedback: a visual image following the auditory sound, seeing the visual image/ reading a textual description simultaneously while hearing the sound. See methods for a full description). In the fifth group, the control, participants still performed the two EyeMusic identification tests, without performing any training in-between. Instead, they had a ~75 minutes of free reading on the computer (i.e., no direct training) between these tests.

The choice of exploring multisensory feedback strategies was motivated by many studies which demonstrated the enhanced efficacy of multisensory over unisensory trainings to improve unisensory perception [17–22] and to diminish response times [23], especially in complex tasks [24] and in cases in which one of the two sensory modalities is weak/degraded [25]. Thus, the inclusion of three different multisensory training groups aimed at investigating whether for this basic SSD training, a multisensory training program would be more effective than a unisensory one, while also allowing the identification of the most efficient multisensory feedback strategy for teaching the use of SSDs. Given the proof-of-concept nature of the current investigation, we chose to deliver audio-visual multisensory stimulations, namely using inputs that can be delivered easily in an online platform. This may of course limit the possibility of extending our results to the blind population, i.e., the main target of SSD training, though only for fully blind users. Indeed, people with visual impairments and some residual vision, or with degenerative visual loss, such as retinitis pigmentosa (RP), which are the majority of visually impaired people [1], could also benefit from audio-visual SSD training. Although use of tactile cues (e.g. [26]) alongside auditory cues, thus creating an audio-tactile multisensory experience would be optimal as they can be used also by blind individuals (see for instance the work of Jicol and colleagues showing advantages towards the combined use of auditory and tactile cues[27]), the use of such settings have their drawbacks. First, the use of tactile information has a lower resolution than auditory and visual cues (tactile bandwidth 100 bits per second [28], audition bandwidth 104 bits per second [29], visual bandwidth of 4.3*106 bits per second [30]). Additionally, the use of a tactile setup is more expensive, and rather complex and too cumbersome to transfer in a remote and free manner, thus potentially hindering the training experience.

Beyond the comparison of multisensory vs. unisensory feedback strategies, our experimental training groups also enable a comparison between perceptual (seeing the visual image) versus descriptive (textual or auditory description of the image) feedback strategies. This comparison can further impact the translational aspect of such SSD online training platform to blind individuals, ultimately hinting on whether descriptive feedback can suffice, and whether its learning outcomes are comparable to those achieved via the perceptual feedback strategy. This comparison is of further interest, as descriptive strategies can be potentially combined with the use artificial intelligence, thus automatically extracting visual content from images and conveying them descriptively to blind users.

The outcomes of this study will shed further light on the effects of multisensory versus unisensory training strategies, and more generally, on the most efficient strategies for learning the basics of SSD. Additionally, and moreover, they will provide guidelines for the implementation of self-training SSD platforms and for future direct testing on the blind and visually impaired populations, ultimately potentially allowing the complementation of one-on-one training and, in turn, possibly facilitating the everyday use of SSDs.

## Methods

### The EyeMusic algorithm

**Fig 1:**
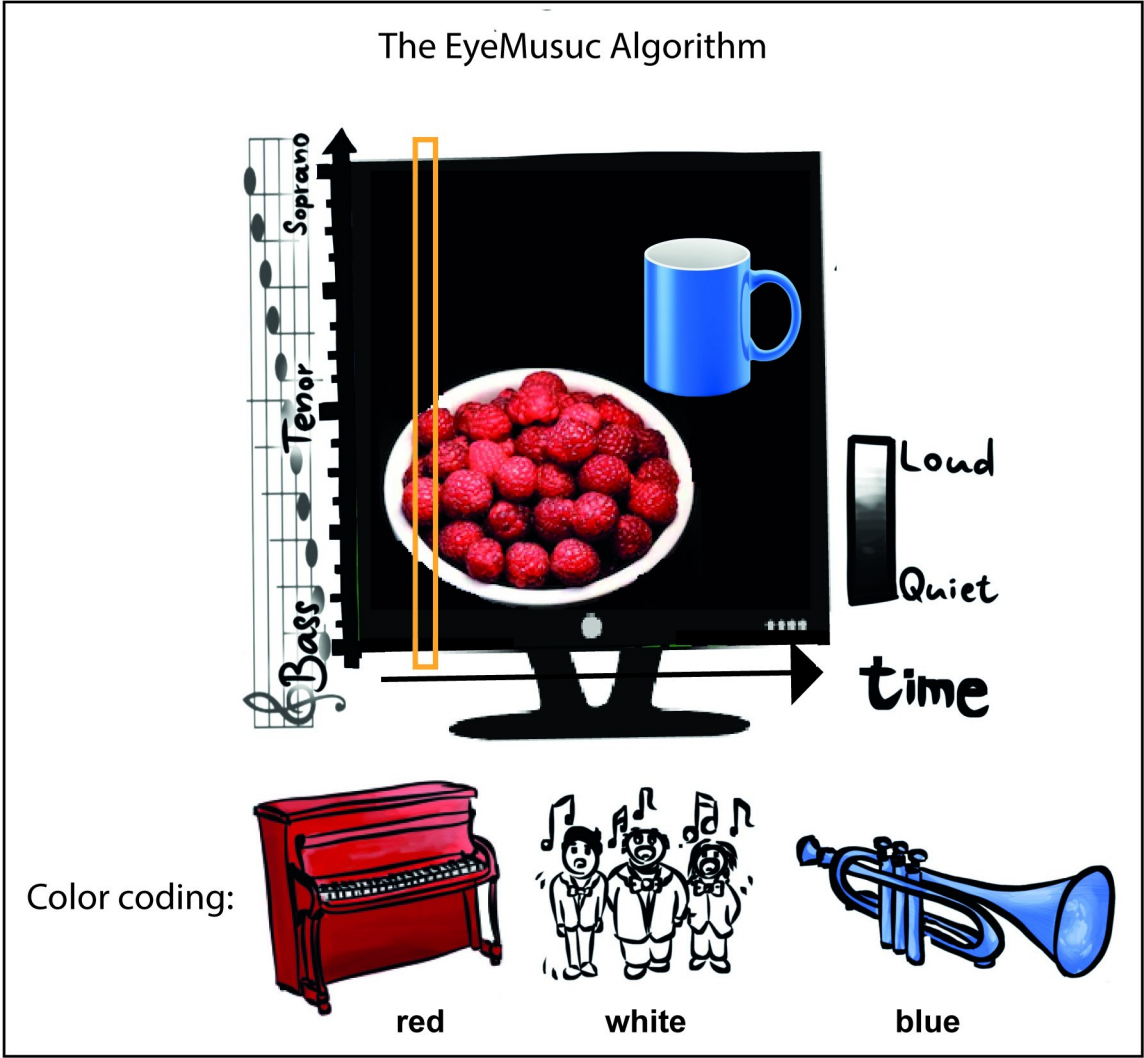
EyeMusic description. The EyeMusic visual-to-auditory SSD transforms visual information into auditory soundscapes. X-axis information is conveyed through time, such that information on the left side of the image is heard before the information on the right. Y-axis information is conveyed through pitch manipulations on the pentatonic scale, such that objects ‘features positioned in the higher portions of the image are sonified with a higher pitch than lower features. Colors are conveyed through timbre variations using different musical instruments. In the current experiment, we used the colors red (piano), white (choir) and blue (trumpet), while silence conveyed black. The orange box, sweeps from left to right, sonifying one column at a time.

In the present study we used the EyeMusic a visual-to-auditory SSD developed in our lab, which transforms whole-visual images into auditory inputs, termed soundscapes, preserving shape, location and even color of the objects in the scene [3]. Specifically, the EyeMusic algorithm down-samples every image to a 30x50 pixels matrix and conveys the x-axis visual information via a left-to-right sweep-line, such that visual features on the left of the image are heard before those on the right. The y-axis positions are conveyed through pitch manipulations, e.g., high-pitched musical notes represent high locations in the image. Different colors are conveyed via different musical instruments (see [3] for full description of the algorithm). In this experiment we used three colors, red, white and blue. Silence is conveyed by an additional forth color, black (see Fig 1).

The EyeMusic has been used successfully for a variety of tasks exploring questions such as sensory-motor information transfer [31], testing visual acuity [32], examining the neural correlates of SSD-presented letters and numbers [33], focusing on particular details of the visual scene and then integrating them into a combined whole [34], and even in practical real world tasks based on shape and color information, such as finding vegetables at the supermarket [13].

### The online version of the EyeMusic

To maximize the usability and distribution of the self-training program, we created an online version of the EyeMusic, which could be accessed via a dedicated website. This website, which includes step-by-step lessons of increasing difficulty for self-training on the EyeMusic SSD, among other EyeMusic related content, was written using ASP.NET MVC technology. One main advantage of an online EyeMusic training platform is that users do not need to install any program to train on the EyeMusic SSD and can train with the algorithm by themselves and at their own pace. Additionally, all the activities of the users are saved automatically on a SQL server database for analyses purposes.

### Participants

Fifty sighted individuals (25 females), aged 26.64±5 years (mean ± SD), participated in this study. The participants were randomly assigned to five groups: Auditory only unisensory feedback (N = 10, 6 females, mean age 24.9+1.57); Interleaved audio-visual, multisensory, feedback (N = 10, 4 females, mean age 25.3+2.9); Simultaneous audio-visual, multisensory, feedback (N = 10, 5 females, mean age 28+4.87); Simultaneous textual description, multisensory, feedback (N = 10, 4 females, mean age 29.5+8.12); Control (N = 10, 6 females, mean age 25.5 +2.45). All participants were naïve to the EyeMusic SSD algorithm as well as to any other SSDs. All participants stated they have normal or corrected-to-normal hearing and vision.

**Fig 2:**
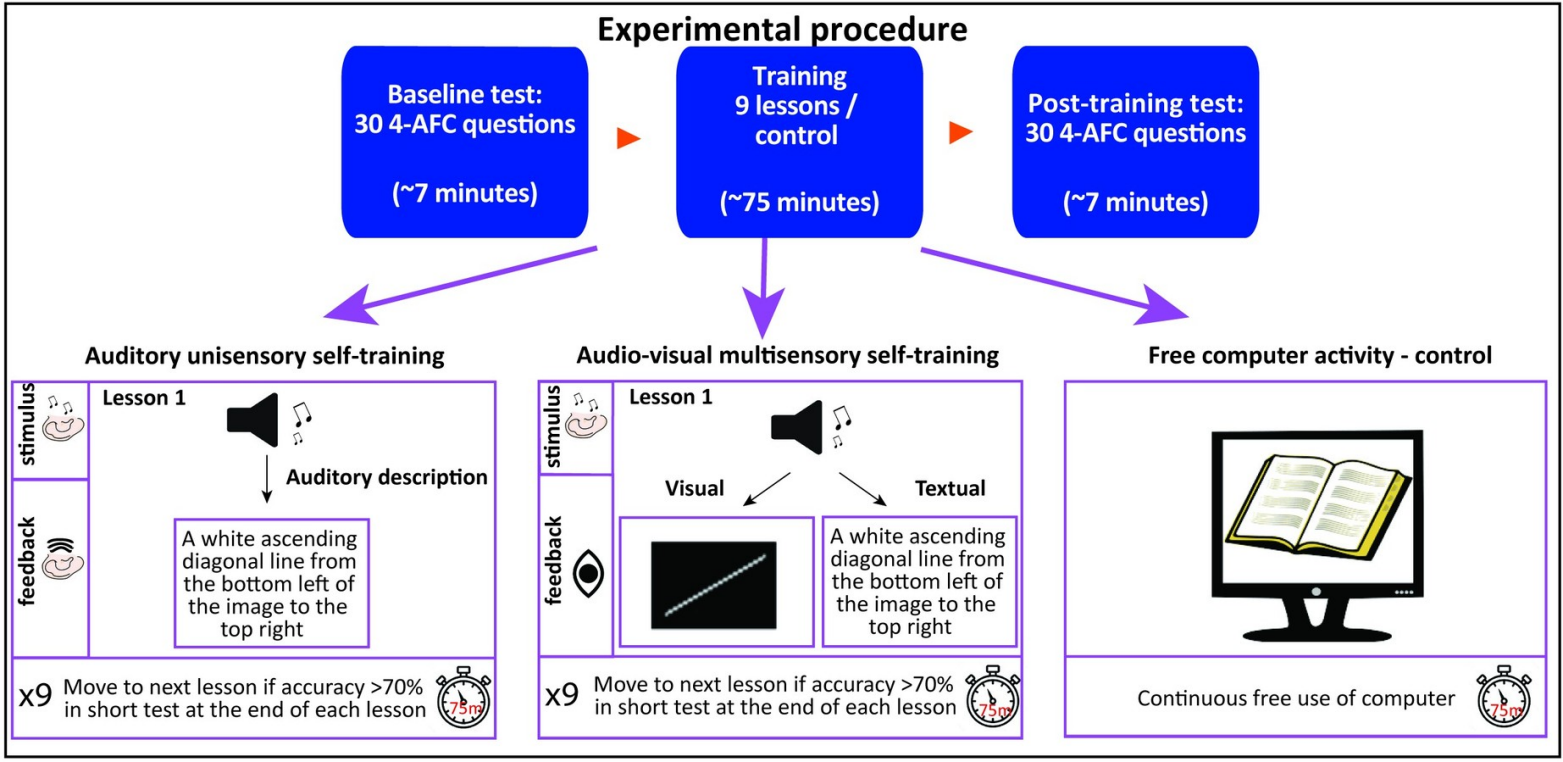
Experimental flow. The experiment included 5 groups of sighted participants, 4 experimental groups, and 1 control group. All participants performed a baseline SSD identification test, and repeated the same test after ~75 minutes. Between tests, the 4 experimental groups participated in a self-learning online training program consisting of 9 step-by-step lessons of increasing difficulty guiding them through the basic principles of the EyeMusic. The feedback method deployed during training to teach the participants to interpret the auditory stimuli of the EyeMusic, varied among groups: 1 Auditory only unisensory group receiving an auditory description of the stimuli after each EyeMusic stimulus; and 3 Audio-visual multi-sensory groups—2 groups perceiving visual images appearing either simultaneously or following the EyeMusic stimuli; 1 group receiving textual descriptions of the stimuli alongside hearing the auditory stimulus; In the control group participants were instructed to free reading from the computer during the ~75 minutes between the two SSD identification tasks.

Participants were compensated for their time and received an additional motivation bonus depending on the lesson-level of EyeMusic they reached at the end of the experiment or, for the control group, on their success rate in the second repetition of the SSD-stimuli identification test, which the other participants performed after EyeMusic training (see details in the next paragraph and see Fig 2). The research protocol was approved by the ethics committee of the Interdisciplinary Center (IDC), Hertzeliya. All participants signed an informed consent form before starting the experiment.

### Experimental setup & procedure

The experiment was conducted on standard PCs (laptop or desktop computers), using standard off-the-shelf headphones, keyboard and mouse.

We developed a self-training program to teach the basic principles of the EyeMusic SSD and tested its efficacy using two identical SSD-stimuli identification tests, interleaved by ~75 minutes of self-training, which comprised a series of 9 step-by-step lessons of increasing difficulty. Before moving on to the next lesson, participants were presented with two self-assessment questions regarding their perceived learning and difficulty of the lesson they just concluded, and were required to answer a short forced-choice SSD-stimuli identification quiz on the material covered during the concluded lesson, aiming at quantitatively assessing the learning of the participants (see Fig 2 for the experimental flow).

Before starting the experimental procedure, participants received a brief verbal explanation on the concept of SSD and on the basic principles of the EyeMusic algorithm. Then, without ever hearing any EyeMusic soundscape, they performed the first SSD-stimuli identification test, which lasted ~7 minutes. Then they started the training procedure which was stopped after ~75 minutes, independently of whether participants completed all the lessons. Finally, the post-training test, which lasted ~7 minutes, started automatically (see Fig 2 and next paragraphs for details on the training procedure). To minimize tiredness of participants, we set the total duration of the experiment, including pre- and post-training tests, to ~90 minutes and this is why the training was automatically stopped after ~75 minutes.

#### Training

The training program included 9 lessons of increasing level of difficulty (i.e. starting with simple single diagonal white lines, adding the blue and red colors, learning other types of lines, combining all types of lines and creating shapes; see supplementary materials Fig 1 for samples of training images for each lesson). At the end of each lesson (except for the last lesson, lesson number 9), participants were asked to self-assess by scaling (1–5: 1 not at all, 5 totally), their perceived learning (“To what extent do you feel that you mastered the materials covered in this lesson?”) and their perceived difficulty (“How difficult was this lesson for you?”). Then participants performed an end-lesson 2-AFC (Alternate Forced Choice) quiz during which they were asked to identify soundscapes conveying the EyeMusic properties that were taught during the specific lesson (10 questions each). Some of the stimuli presented in these tasks were taken from the training lesson they have just completed, while the rest, at least 60%, were novel to the participants (untrained), though still testing the concepts learned during that specific lesson. At the end of the quiz, participants received their overall accuracy level, but were not informed which questions they answered correctly/wrongly. If in the end-lesson quiz, they reached a success rate of at least 70% (i.e. they answered correctly at least 7/10 questions), they moved to the next level. Participants who did not reach this level of accuracy, repeated both the lesson and the related quiz (the quiz’s questions and order did not change between repetitions).

This experiment included four training groups varying in the type of feedback they received on the auditory SSD stimuli they heard during training, and a fifth, control group. Specifically, the four training groups varied in the following manner: 1*) Auditory only* u*nisensory feedback (auditory)*: in this group participants heard each auditory EyeMusic soundscape, and then, for feedback, it was followed by a detailed auditory verbal description of it. 2) I*nterleaved audio-visual*, *multisensory*, *feedback (interleaved audio-visual)*: in this group participants heard each auditory EyeMusic soundscape and then, for feedback, they saw on the screen the visual image it conveys. 3) *Simultaneous audio-visual*, *multisensory*, *feedback (simultaneous audio-visual)*: in this group participants heard each auditory EyeMusic soundscape and then, for feedback, they heard it again while seeing the matching visual image. 4) *Simultaneous* t*extual description*, *multisensory*, *feedback (textual)*: in this group participants heard each auditory EyeMusic soundscape, and then, for feedback, they heard it again while reading its textual description.

In all feedback groups, participants heard each auditory soundscape repeatedly, until they pressed a button to end the auditory soundscape repetition and receive its description (i.e., feedback). In the auditory feedback and the interleaved audio-visual feedback groups, the feedback (the auditory description or the visual image, respectively) was presented alone (i.e. without hearing the auditory soundscape it described). After receiving the description, following a button press, they heard the soundscape again for three more times, and then could choose whether to continue on to the next stimulus or to receive the stimulus description again. In the simultaneous audio-visual feedback and the textual feedback groups, the feedback was presented while hearing the auditory soundscape which was repeated twice. Then, the auditory soundscape was heard once again alone and then participants could choose whether to continue on to the next stimulus or receive the stimulus description again.

After ~75 minutes from the beginning of the training, participants were automatically directed to the post-training SSD identification test. If participants were in the middle of an end-lesson quiz, the transfer to the final test occurred only after completion of the current end-lesson quiz.

#### Control group

Participants in this group performed the pre-training SSD identification test as the other groups, then they had ~75 minutes of free reading on the computer, at the end of which they repeated the SSD identification test, without any training on the EyeMusic. During free reading they were instructed to read anything they wanted with the only constraint of not reading anything related to sensory substitution devices.

#### Pre- post-training SSD identification tests

The pre- and post-training tests were identical. They included 29 4-AFC questions on 29 different EyeMusic stimuli. For each question, participants heard only the soundscape while reading the related question on the screen. The soundscape was repeated until a response was provided, with a time-limit of 45 seconds (i.e., reaching the time limit with no response was considered an incorrect response and the program automatically moved on to the next question). The original test had 30 questions but one of those questions was removed from analysis due to technical issues (see supplementary materials Fig 2 for a complete list of the images and questions). To investigate generalization properties, most of the stimuli of the SSD identification test included novel, untrained stimuli (83% novel stimuli), alongside few trained stimuli, namely already presented during the lessons (17% of stimuli).

#### Final survey

After the post-training test, all participants filled out a survey about the training. These questions regarded participants’ musical background and their subjective feeling about the training process.

## Results

**Fig 3:**
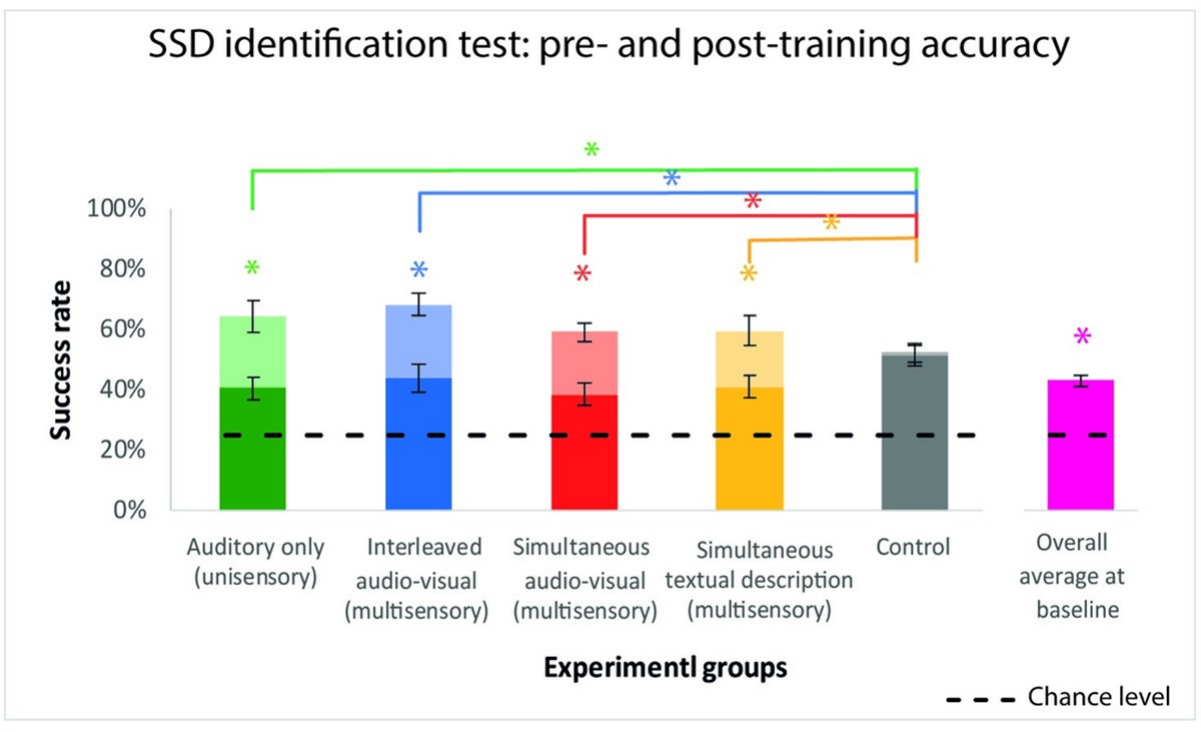
Pre- and post-training accuracy in the SSD identification test for all experimental groups. Baseline average accuracy level in the pre-training test is depicted in the bottom part of each stacked bar. Average accuracy in the post-training test is depicted in the top part of each stacked bar (shaded colors). First, when comparing accuracy in the pre-training test, no difference was observed between experimental groups (Kruskal-Wallis p-value = 0.2). Pooling the baseline measurement amongst all participants from all experimental conditions (43% ± 12%, pink bar) was significantly higher than a chance level of 25% (two-sample t-test, unequal variance, p< 0.00001, asterisk on top of the bar). Importantly, post-training accuracy rate in each of the four training groups was significantly higher than their accuracy in the pre-training SSD identification test (Wilcoxon sign-rank, auditory only (unisensory) p-value = 0.004; interleaved audio-visual (multisensory) p-value = 0.002; simultaneous audio-visual (multisensory) p-value = 0.002; simultaneous textual description (multisensory) p-value = 0.0098). This was not the case in the control group (Wilcoxon sign-rank, p-value = 0.9) (asterisks on top of the stacked bars). Additionally, when calculating the improvement-in-accuracy index as the difference in accuracy between pre- and post-training tests (shaded bar graphs), a significant effect emerged among experimental groups (Kruskal-Wallis p-value = 0.006). Post-hoc Wilcoxon rank-sum analysis revealed that this was driven by a significant difference between the control condition and all four training conditions (auditory only (unisensory) vs. control p-value = 0.006, interleaved audio-visual (multisensory) vs. control p-value = 0.001, simultaneous audio-visual (multisensory) vs. control p-value = 0.002, simultaneous textual description (multisensory) vs. control p-value = 0.03), while no other differences were significant (all p-values >0.33). Note that in all the stacked bars depicted here, error bars show the standard error.

To evaluate whether all of our participants started out with the same baseline accuracy level, we performed a Kruskal-wallis test on the accuracy in the pre-training test of all participants in the different training conditions (auditory = 40%±11% (average ± SD), interleaved audio-visual = 44%±15%, simultaneous audio-visual = 38%±12%, textual = 41%±11%, control = 52%±11%). Results confirmed that, as expected, there was no significant difference in the baseline accuracy level among groups (Kruskal-wallis, p = 0.2) (see Fig 3).

Furthermore, we wanted to check whether participants’ performance at baseline, i.e., before any EyeMusic training, would be higher than the chance level of 25%. Since there was no significant difference between baseline accuracy among groups, we pooled together all the results from the pre-training tests, irrespective of the group. Results showed that participants, before any EyeMusic training, performed significantly above the chance level (two-sample t-test, p < 0.00001, FDR correction, alpha = 0.05, N = 20) (see Fig 3).

Additionally, we were interested in investigating whether the different training conditions significantly increased the accuracy of participants in the post-training SSD identification test and whether there was a difference in improvement depending on the training strategy used. First, we found that in each of the training groups, participants’ post-training average accuracy was significantly higher than the baseline average accuracy obtained in the pre-training test (average post-training accuracy: auditory = 64%±13%, p = 0.0039; interleaved audio-visual = 68%±11%, p = 0.002; simultaneous audio-visual = 59%±12%, p = 0.002; textual = 60%±12%, p = 0.0098; all p-values were calculated using the Wilcoxon sing-rank test, all survived FDR correction, alpha = 0.05, N = 20). This was not the case in the control group (control = 52%±18%, p = 0.9). Additionally, we wanted to investigate whether there were differences in efficacy among the different feedback training strategies. To this aim, we calculated the improvement-in-accuracy index as the difference in accuracy between pre- and post-training SSD identification tests (auditory = 24%±17%, interleaved audio-visual = 25%±12%, simultaneous audio-visual = 21%±9%, textual = 19%±16%, control = 1%±10%). We then performed a Kruskal-Wallis test with this index as a dependent variable, comparing all 5 experimental conditions. This yield a significant effect (p = 0.006, survived FDR correction, alpha = 0.05, N = 20). Post-hoc Wilcoxon rank-sum analysis revealed that this effect was driven by the significant difference between the control condition and all other 4 training conditions (auditory vs. control p = 0.006, interleaved audio-visual vs. control p = 0.001, simultaneous audio-visual vs. control p = 0.002, textual vs. control p = 0.03, all survived FDR correction, alpha = 0.05, N = 20), while no other differences were significant (all p-values >0.33) (see Fig 3).

**Fig 4:**
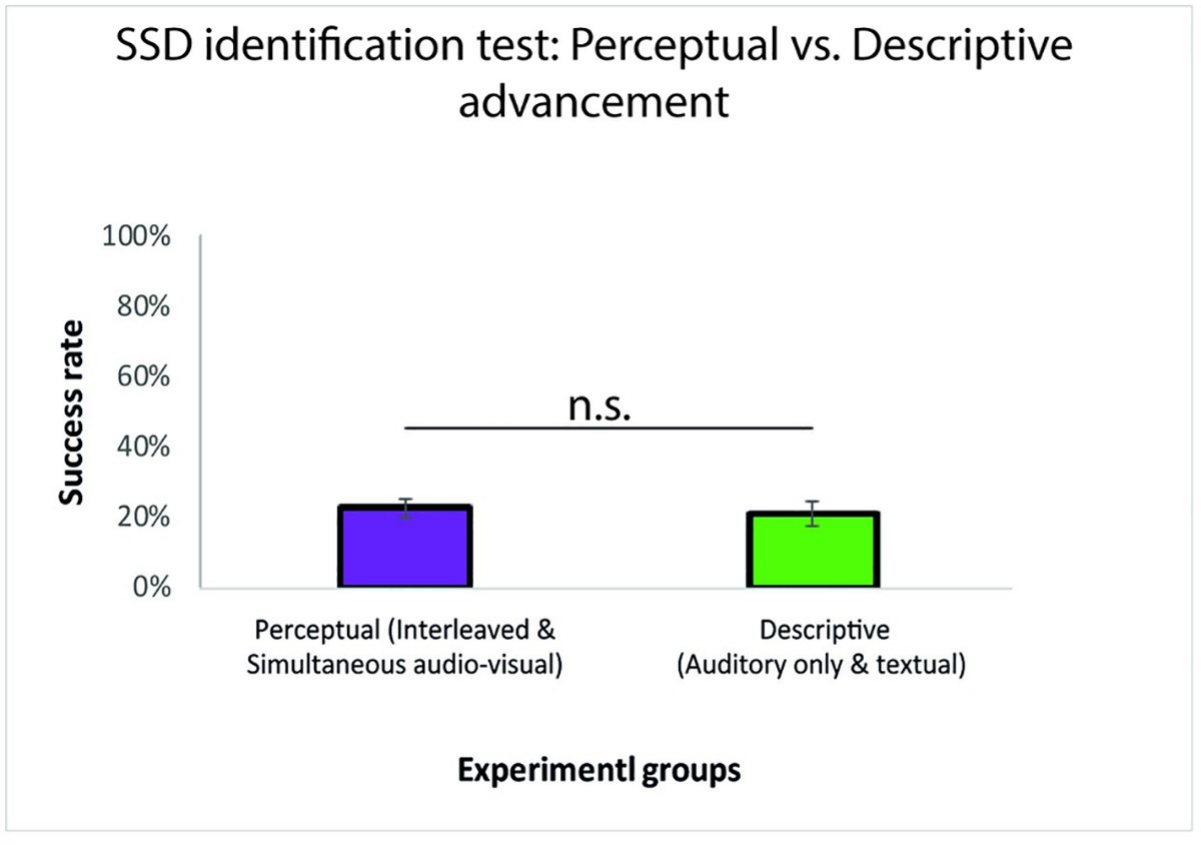
Improvement in accuracy between pre- and post-training SSD identification test for perceptual and descriptive training groups. When pooling together the improvement-in-accuracy index for all perceptual training strategies (interleaved and simultaneous audio-visual multisensory), and the improvement-in-accuracy index for the descriptive training strategies (auditory only unisensory, and audio-visual textual descriptive), no significant difference was found (rank-sum, p = 0.99). Note that the error bars show the standard error.

Finally, to investigate whether learning was modulated by the perceptual vs. descriptive nature of feedback strategies, we pooled the improvement-in-accuracy index across the perceptual (interleaved and simultaneous multisensory audio-visual) and descriptive (auditory only and textual) feedback training strategies (perceptual = 23%±11%, descriptive = 21%±16%). No significant difference was found between these two training strategies (Wilcoxon rank-sum, p = 0.99) (see Fig 4).

**Fig 5:**
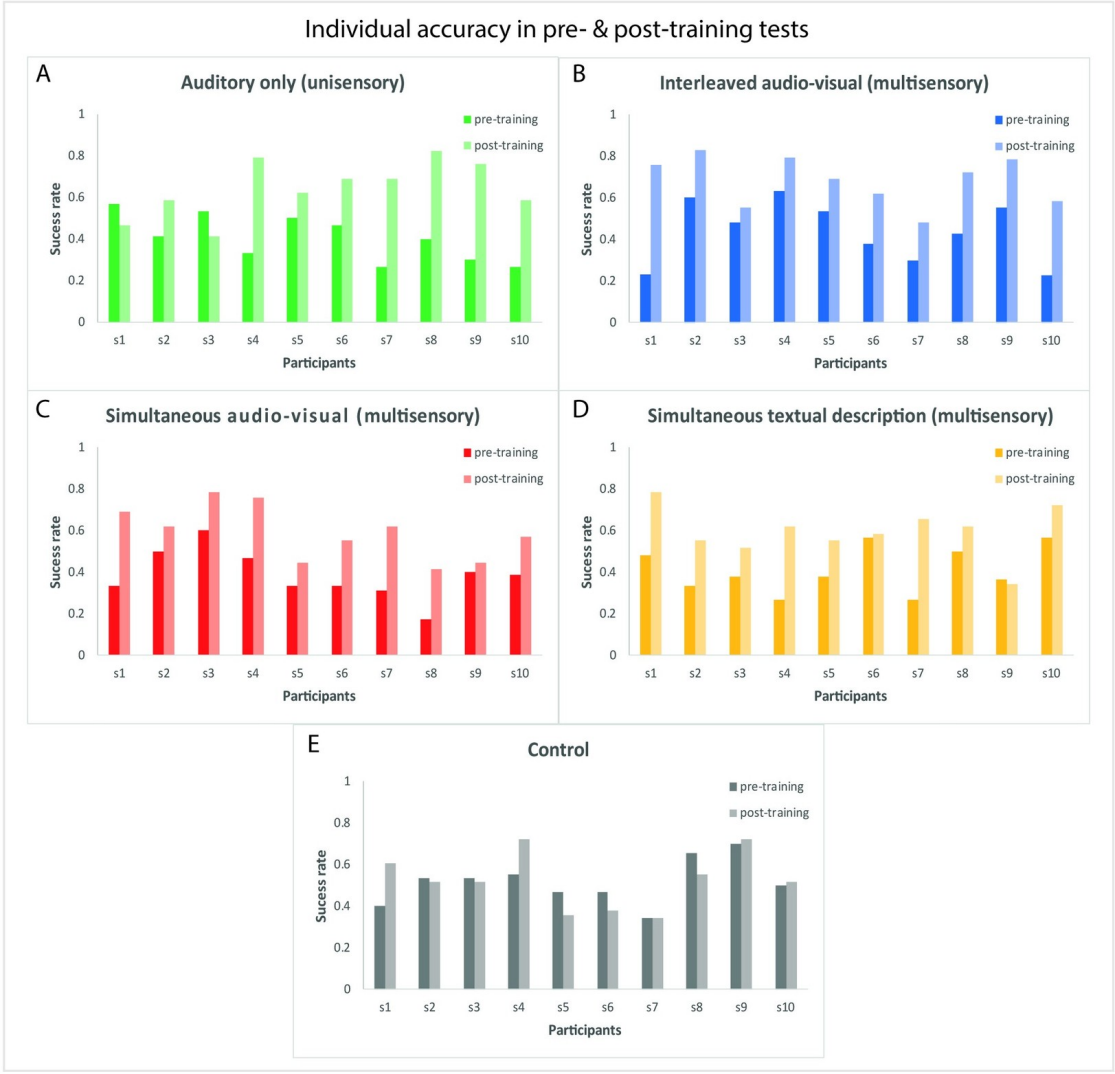
Individual accuracy in pre- and post-training SSD identification test, separated for each experimental group. Each graph shows the success rate of a single participant in pre-training (dark bars) and post-training SSD identification tests (light bars). **A. Auditory only (unisensory) training group:** 8 out of 10 participants improved their success rate in the post-training test compared to their pre-training performance. **B. Interleaved audio-visual (multisensory) training group:** all participants improved their success rate in the post-training test compared to their pre-training performance. **C. Simultaneous audio-visual (multisensory) training group:** all participants improved their success rate in the post-training test compared to their pre-training performance. **D. Simultaneous textual description (multisensory) training group:** 9 out of 10 participants improved their success rate in the post-training test compared to their pre-training performance (note that 1 out of these 9 participants showed a very minimal improvement in the post-training test). **E. Control group:** 4 out of 10 participants improved their success rate in the post-training test compared to their pre-training performance (note that 2 out of these 4 participants showed a very minimal improvement in the post-training test).

However, we observed some interesting tendencies suggesting that multisensory training conditions tended to outperform the auditory unisensory one. For instance, when looking at the individual participants’ results in post- versus pre-training tests, we observed that all participants in the interleaved audio-visual and simultaneous audio-visual multisensory training groups showed an improvement between the two tests, while both for textual and for the auditory unisensory feedback condition, such improvement did not happen for all participants (see Fig 5).

Additionally, in all multisensory training groups (interleaved audio-visual, simultaneous audio-visual and textual), participants in the ~75 minutes of training went further in the online-training step-by-step lessons. The median lesson the participants in these groups reached in the training program, was the 7^th^ lesson. In the unisensory group, the median lesson participants reached, was the 6^th^ lesson (auditory = 6±1 (median lesson number ± MAD), interleaved audio-visual = 7±0.5, simultaneous audio-visual = 7±0.5, textual = 7±1; see Table 1 for the number of participants which participated in each end-lesson quiz separately for the four experimental groups). The individual number of successfully completed lessons, significantly correlated with participants’ success rate in the post-training test at the end of the training program (R = 0.47, p = 0.0019, FDR correction, alpha = 0.05, N = 20).

**Table 1:** End-lesson number of participants and quiz repetitions.

	End-lesson quiz: Number of participants and quiz repetitions															
	1		2		3		4		5		6		7		8	
	No. participants	No. repetitions	No. participants	No. repetitions	No. participants	No. repetitions	No. participants	No. repetitions	No. participants	No. repetitions	No. participants	No. repetitions	No. participants	No. repetitions	No. participants	No. repetitions
**Auditory only (Unisensory)**	10	0	10	1	9	0	9	2	7	0	7	0	4	1	1	0
**Interleaved audio-visual (multisensory)**	10	0	10	0	10	0	10	3	10	0	10	0	8	4 (3 sub.)	3	0
**Simultaneous audio-visual (multisensory)**	10	1	10	2 (1 sub.)	10	0	10	1	10	3	10	9 (6 sub.)	8	3	3	0
**Simultaneous textual description (multisensory)**	10	3 (2 sub.)	10	2	10	2	10	2	10	0	10	10 (5 sub.)	8	2	4	0

The table shows for each group the number of participants who participated in the end-lesson quiz, and how many times the quiz was repeated (total amount of quiz repetitions and in parentheses the number of participants who repeated the quiz). In all three multisensory training groups participants got to more advanced lesson within the ~75 minutes of training as opposed to the unisensory group (auditory = 6±1, interleaved audio-visual = 7±0.5, simultaneous audio-visual = 7±0.5, textual = 7±1). Participants from both the auditory only unisensory group, and the interleaved audio-visual groups, had less quiz repetitions.

Another measure of efficacy of the different training programs, is the performances in the end-lesson identification quiz (i.e., whether participants reached an accuracy level < 70% and thus had to repeat a given lesson and the related identification quiz). To quantify this information, we calculated the average ratio of quiz repetition in each training group. Specifically, first, for each participant we calculated the average of quiz repetitions throughout training. Then, we averaged those ratios to obtain an average ratio of lessons’ repetitions for each group. Results show that this ratio tended to be lower for participants from the auditory and interleaved audio-visual groups, indicating they had to repeat less lessons than participants in the simultaneous audio-visual and textual groups (auditory only = 1.08±0.16, interleaved audio-visual = 1.1±0.13, simultaneous audio-visual = 1.2±0.23, textual = 1.3±0.39). The individual repetition ratio significantly correlated with participants’ success rate in the post-training test (R = -0.54, p = 0.0003, surviving Bonferroni correction, alpha = 0.05).

**Fig 6:**
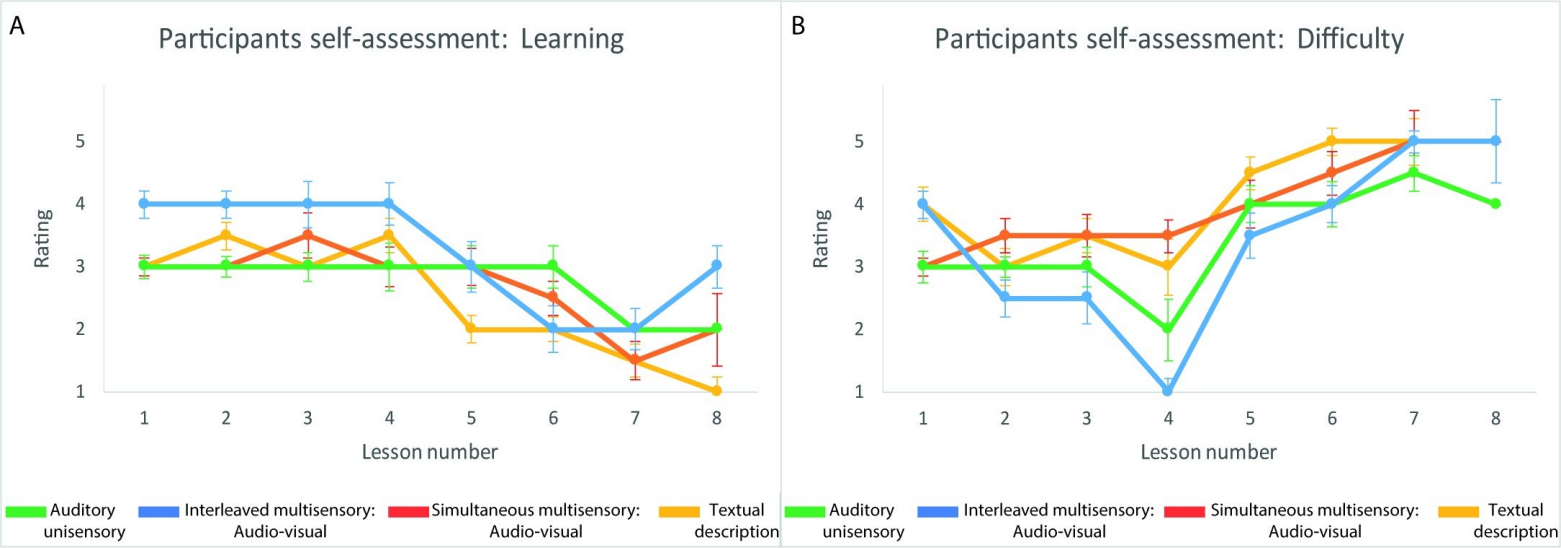
Participants self-assessment. Following each training lesson, and before the end-lesson quiz, participants rated in a 1–5 scale two self-assessment questions: 1) their subjectively perceived learning of the material presented in each lesson, 2) how difficult they subjectively rated each lesson. **A. Learning self-assessment:** The median of self-evaluation of learning, was highest for participants from the interleaved audio-visual (multisensory) group (blue), followed by participants from the auditory only (unisensory) group (green). **B. Difficulty self-assessment:** The median of self-evaluation of difficulty, was lowest for participants from the interleaved audio-visual (multisensory) group (blue), followed by participants from the auditory only (unisensory) group (green). Note that all error bars here represent MAD.

Nicely, also the results of the scaling-questions regarding the self-perception of learning and difficulty presented at the end of each lesson, show a similar tendency. Specifically, after each lesson, and before entering their responses in the end-lesson quiz, participants were asked to scale (from 1 to 5) their perceived learning level regarding the stimuli they were exposed to in each lesson, and how difficult they perceived the lesson. When plotting the median responses provided by participants separately for each lesson and training group, one can observe a tendency of participants from the interleaved audio-visual training group, to scale higher their learning level, alongside lower scaling of their perceived difficulty (see Fig 6).

## Discussion

Our results showed that for all four training conditions, our online-training methods were successful in significantly improving accuracy in the post-training test compared to the pre-training test. This was not the case in the control group, in which the “post-training” success rate was not significantly different than the accuracy rate in the “pre-training” test.

The significant improvement in the success rate in the post-training test of all participants from all training conditions is even more impressive if one considers that over 83% of the stimuli included in the SSD identification test were novel and not learned during the training phase. This excludes the possibility that such post-training improvement is due to memory effects. Furthermore, it supports the generalization ability of SSDs users to perceive untrained stimuli, though similar to the trained ones (see also [35–37]). This result is a first step suggesting the feasibility of SSDs for everyday use, where one often encounters new stimuli belonging to known categories. It is important to note though, that differently from actual real-world use, here we presented simple geometric shapes, thus future studies will need to replicate generalizability of learning within richer and more ecological training environments (see also section dynamic vs. static training below).

Participants success at baseline demonstrates the intuitiveness of the basic principles of the EyeMusic algorithm and further strengthens previous findings obtained with other visual-to-auditory SSDs algorithms, reporting intuitive learning in the initial stages of SSD-related trainings [15, 38–40]. Note that this probably depends on the fact that many visual-to-auditory SSDs are based on known cross-modal correspondences between vision and audition (e.g. high positions in space correspond to high-pitch sounds [41–44]), thus facilitating the understanding and the learning of the features of SSD algorithms [15].

### Multisensory vs. unisensory training

Contrary to the wealth of evidence reporting better learning during multisensory than unisensory stimulations [17–22, 45], our results showed no significant difference between multisensory and unisensory training approaches. Specifically, we did not observe any significant difference among the improvement levels reached at the end of the four training programs in the post-training test. Nonetheless, we observed some tendencies for an advantage of multisensory audio-visual over auditory unisensory training strategies. For instance, when looking at individual success rate in the pre- and post-training SSD identification test, we observed that in the interleaved audio-visual and simultaneous audio-visual multisensory training groups, 100% of the participants improved their success rate in the post-training test compared to their score in the pre-training test, while in the textual group, and especially in the auditory unisensory training group, such improvement was less consistent across participants (see Fig 4). Additionally, when considering how many training lessons participants successfully completed before the training was stopped after ~75 minutes, we observed that participants from all three multisensory groups (interleaved audio-visual, simultaneous audio-visual and textual) tended to complete more lessons of our self-training program. Interestingly, we showed that the individual number of successfully completed lessons significantly correlated with the overall success rate achieved in the post-training test. This latter result, in turn, corroborates the conclusion that the multisensory approach tended to be more effective than the unisensory one. Note however, that this result might be at least partially due to differences in the speed of processing between vision and audition. Indeed, the perception of the visual feedback (received in all three multisensory training groups) is quicker than the auditory one (received in the unisensory training), thus potentially making the advancement in the entire training program faster.

Additionally, we observed that participants in the interleaved audio-visual and auditory unisensory training groups tended to repeat overall less lessons (i.e., more often reached an accuracy >70% in the end-lesson quiz in their first attempt) compared to participants in the simultaneous multisensory and reading groups. Moreover, the individual repetition ratio significantly correlated with the success rate in the post-training test. This suggests, in turn, that the auditory and audio-visual training programs tended to be more effective in teaching the basics of the EyeMusic. Nicely, these results fit well with participants’ end-lessons self-assessment, where we observed a tendency of participants from the interleaved audio-visual and auditory unisensory groups to scale their learning as higher, and the level of difficulty as lower. Thus, both these tendencies together suggest that an interleaved training approach, whether unisensory or multisensory, seems to be more efficient than simultaneous training approaches for the initial stages of SSD training. This result is also in line with previous evidence showing the effectiveness of interleaved multisensory training in improving sound localization of deafened ferrets [46]. The effectiveness of an interleaved training strategy is probably due to the fact that this approach forces participants to focus more on the novel sensory information which is presented alone, compared to simultaneous training strategies where the focus of attention towards the novel sensory information might diminish in favor of the supplementary, more familiar sensory input.

All the aforementioned results together, highlight a tendency for the interleaved audio-visual approach to be the most efficient feedback strategy during training (i.e., highest number of completed lessons; lowest number of lessons’ repetition ratio and better end-lesson self-assessment scores), even though at a pure statistical level, we did not find any difference among the various training approaches. One possibility is that our group sizes were too small to catch potential significant differences in this regard. Another possibility for this lack of a statistical advantage of multisensory strategies is that we trained here the basic principles of the EyeMusic SSD, using relatively simple stimuli (i.e., lines and simple shapes). Indeed, the inverse effectiveness rule which is used to determine the effectiveness of multisensory stimulations on perception, postulates that multisensory enhancement has higher efficacy in perceptual situations in which one of the two sensory inputs is either deteriorated or very complex [47, 48]. It might be that in the case of basic SSD properties, the auditory signal is not complex enough to significantly benefit from additional multisensory inputs. This option is further strengthened by the average accuracy at baseline, without any SSD training, in identifying SSD stimuli, which resulted significantly higher than chance level.

Currently, the training duration was relatively short, ~75 minutes. We choose a relatively short training duration since we were interested in investigating the efficacy of relatively quick self-training programs. A short training program is important, as many potential SSD users are reluctant to use these devices due to the long training required for mastering them. Overall, our current findings show that self-training on the initial learning phases of an SSD algorithm is indeed possible and can efficiently occur relatively quickly. However, in our study, participants’ accuracy level after training was only at around 63%, namely still far from ceiling (see [15] for similar findings). Thus, we assume that with longer training, participants’ overall accuracy rate could further increase. Additionally, longer training programs will enable to introduce more complex SSD soundscapes, making the learning more useful for real-life tasks. Possibly, with longer training and a bigger sample, differences in the final outcomes among the various training strategies will become more apparent and might unravel an advantage of multisensory over unisensory approaches. We think that an initial shorter and entirely autonomous training program, might serve the crucial function of intriguing the users, ultimately encouraging them to further train on the device with a longer training program aimed at achieving more benefits from the use of SSDs.

### Testing sighted participants as a proof-of-concept for the efficacy of SSD self-training

As visual-to-auditory SSDs are mainly aimed at being assistive technology for the blind and visually impaired population to convey visual information and ultimately maximize their independent interactions with the environment, this stake-holder population is also the final target of the current self-training SSD program. Here however we tested only sighted individuals, as a proof-of-concept for the feasibility of this approach and as a first step towards the identification of the most suited training strategy. Specifically, testing the sighted population allowed us to easily create a self-training platform which was able to deliver multisensory training lessons based on audio-visual pairing, which is easily and freely available in an online platform. Implementing multisensory training for blind individuals would have entailed the involvement of audio-tactile inputs, requiring an additional hardware component (i.e., to deliver tactile SSD stimulations), which both raises costs and is harder to adapt to an online platform as it would require constant maintenance. It is important though to note, that multisensory audio-visual approaches can potentially impact the rehabilitative aspects of visually impaired individuals, for instance, individuals with residual vision, or with degenerative visual loss (see for instance [49, 50] suggesting the coupling of SSDs with sight restoration approaches). Our current results on the sighted population show that for this initial stage of SSD training, the unisensory and multisensory, perceptual and descriptive training methods were equally efficient. This suggests that a self-training program tailored to the blind population using a unisensory descriptive auditory feedback training strategy to teach them the basic principles of the visual-to-auditory EyeMusic SSD, might be effective.

However, the fact that we used only sighted participants is obviously also a limitation for the translational aspect of this work. Note though, that our platform has been already designed in a fully accessible manner, thus making the testing of the blind and visually impaired populations relatively straightforward to implement in future works. Furthermore, the fact that we did not find any significant difference in the learning outcomes following perceptual (interleaved and simultaneous audio-visual) and descriptive (auditory-only textual reading) feedback training strategies, strengthens the hypothesis that audio-only descriptive feedback might be effective for conveying basic SSD-transformed visual content to both congenital and late blind visual-to-auditory SSD users. Indeed, the visual content delivered in the current training programs relates to visual concepts that are familiar also to congenital blind individuals (e.g. size, line orientations, simple shapes are commonly perceived by blind people via the tactile modality). We hypothesize auditory description might suffice also for successfully conveying color: although obviously fully blind individuals cannot perceive color via other sensory modalities, the concept of color is semantically familiar to them (i.e., they are constantly exposed to colors during linguistic interactions). Importantly, the current training only requires associating specific colors with specific sounds, thus remaining in a conceptual domain. Therefore, we predict that such an online self-training program will be successful also in blind individuals. Note that, in addition, previous studies comparing the use of SSDs between blind and sighted participants have shown that SSD learning can be effective in both groups [5, 32, 51–54]. Therefore, future studies directly testing the applicability of such online training in blind individuals could also add to this latter literature. Potentially, the online (and free) nature of this self-training we developed, and its possible use without sighted assistance, will significantly increases its availability to the blind community, a problem often limiting previous training initiatives which required travel, and the high cost of the offered training programs [9]. Such an online platform, together with further development of the training programs, might then succeed in loosening the training bottleneck and in spreading the use of SSDs among blind people. For instance, this online self-training can be extended to include also more active training, which can potentially boost the users performance [55–57].

Finally, while the primary use of most SSD transformations is for sensory rehabilitation, they can also be used potentially for sensory augmentation. In such use cases, sighted users learning a visual-to-auditory tranformation (e.g. where the visual information might be coming from a heat sensor) might consider using visual input as part of their training, and our results are relevant to these use cases as well.

### Dynamic vs. static training

The training used here, in the self-training program, is the basic and most common method, namely using a series of static stimuli [36, 58, 59]. However, while effective, as demonstrated here, this type of learning shows limitations when aiming at training for real world scenarios, and can quickly become boring for the users ultimately harming users’ motivation. Many evidence suggest that adding more dynamic aspects to training environments boosts learning and also increases users’ enjoyment [60–66]. Thus, the next steps of this training program should include dynamic scenarios such as games, and tailored virtual environments [67], which we are currently testing. Finally, the last stage of SSD training will involve full immersive use of SSDs in the real world. Note that perceiving objects or full scenes via SSDs is a very complex task, requiring dedication and often personalized feedback. Thus, we propose that for promoting the use of SSDs in real-life, the final training solution might probably entail a combination of an initial relatively-short and entirely self-monitored training program, followed by a longer training program carried out in a mixture of self-learning and supervised training with an instructor (and potentially in the future using artificial intelligence allowing the individualization of the training content and strategies based on the user’s performances). Potentially, this combined training approach will promote the overall and everyday use of SSDs. These training programs should keep in mind the blind target population, including congenitally blind individuals, to whom some of the visual concepts, such as depth can be novel. Special thought needs to be given towards the translation of these concepts to their available sensory experiences (see the work of Renier & De Volder regarding depth perception via SSDs [68]).

Another important aspect of our self-training approach is the possibility to measure training parameters, and to control for the exact training history and/or training level of participants while using the SSD. Despite numerous imaging studies which have shown the recruitment of the deprived visual cortex by auditory SSD inputs after training (cross-modal plasticity) [69–73], there has yet to be a sufficiently systematic exploration of the neural correlates of the different stages of this cross-modal recruitment, based on the level of proficiency in using SSDs. This tool can be crucial in providing controlled parameters for this exploration.

## Conclusions

In the current paper we presented a proof-of-concept study demonstrating the feasibility of self-training to learn basic principles of visual to auditory SSDs algorithms in the sighted population. We also showed that at this initial stage of learning, auditory unisensory and multisensory, audio-visual, training methods are equally efficient, even though we also report a tendency for the interleaved audio-visual training strategy to be the most efficient. Interestingly, we showed that the performance in the pre-training SSD identification task was above the chance level, even without any training. This suggests that some aspects of the EyeMusic visual-to-auditory SSD are so intuitive that can be interpreted even without any specific training.

Self-training of sighted participants on the perception of basic stimuli is the first step upon this path. Our next steps will include testing this approach with blind individuals, alongside exploration of online self-training advanced scenarios such as dynamic games, images from the real world and tailored virtual training environments. This work has the potential of contributing to a widespread use of SSDs among blind and visually impaired individuals, by creating a self-training SSD setup easily available or by complementing the existing programs with sighted instructors, enabling blind users to practice the use of SSDs also independently.

## Supporting information

S1 Fig Stimuli sample: Examples of the different stimuli presented in the different step-by-step lessons. (TIF)

S2 Fig Pre-post training identification test–list of stimuli task, questions and the correct answer. (TIF)

S1 Dataset Data–an excel file including the experimental data. (XLSX)
