# Correction: A self-training program for sensory substitution devices

**DOI:** 10.1371/journal.pone.0287802

**Published:** 2023-06-23

**Authors:** Galit Buchs, Benedetta Heimler, Menachem Kerem, Shachar Maidenbaum, Liraz Braun, Amir Amedi

The second author’s name is spelled incorrectly. The correct name is: Benedetta Heimler. The correct citation is: Buchs G, Heimler B, Kerem M, Maidenbaum S, Braun L, Amedi A (2021) A self-training program for sensory substitution devices. PLoS ONE 16(4): e0250281. https://doi.org/10.1371/journal.pone.0250281
