# Supplementary figures and images for: A self-training program for sensory substitution devices
Source: PLoS One. 2021 Apr 27;16(4):e0250281. doi: 10.1371/journal.pone.0250281 (PMC8078811; doi:10.1371/journal.pone.0250281)

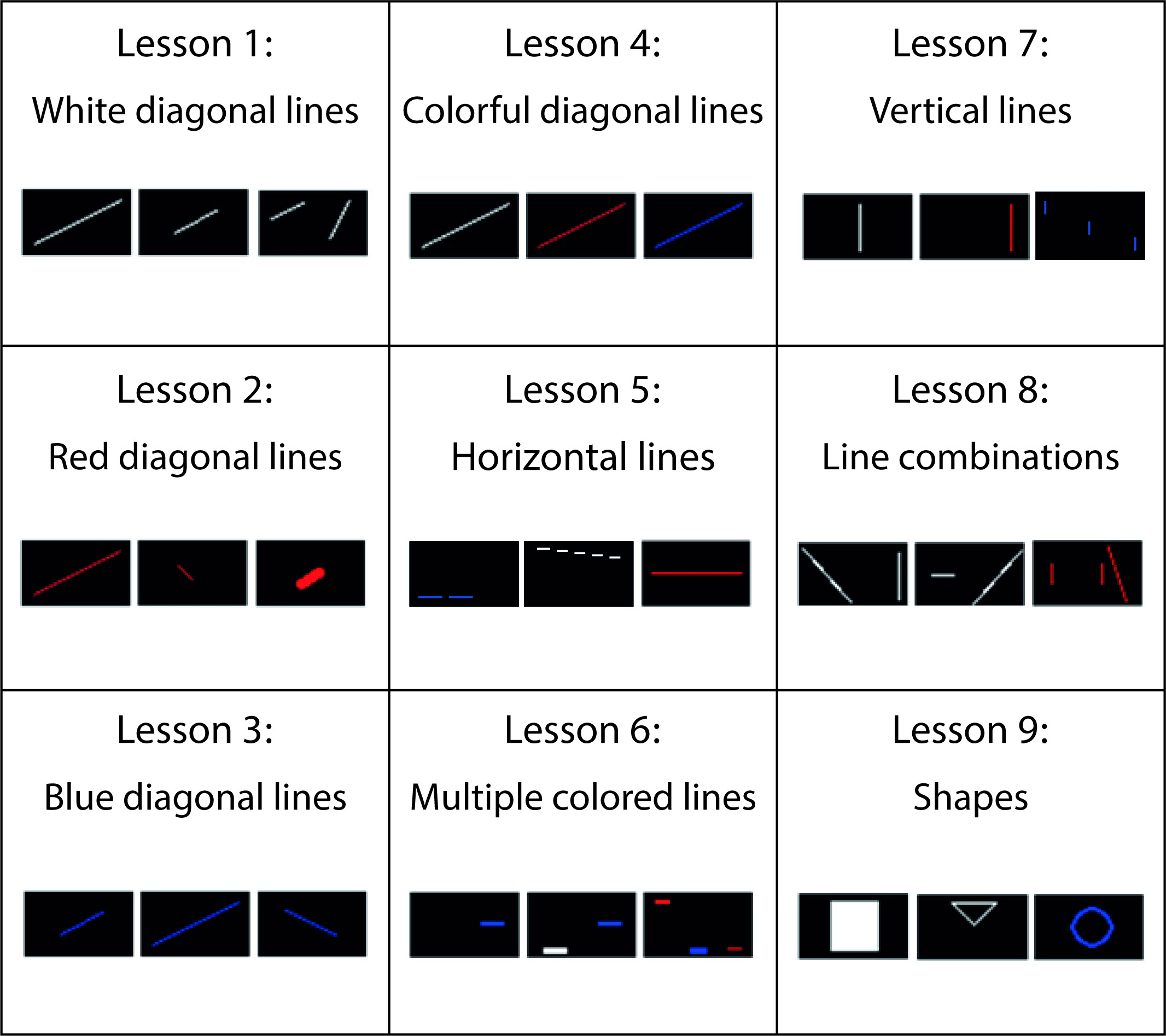

Supplement: S1 Fig — (TIF) [file pone.0250281.s001.tif]

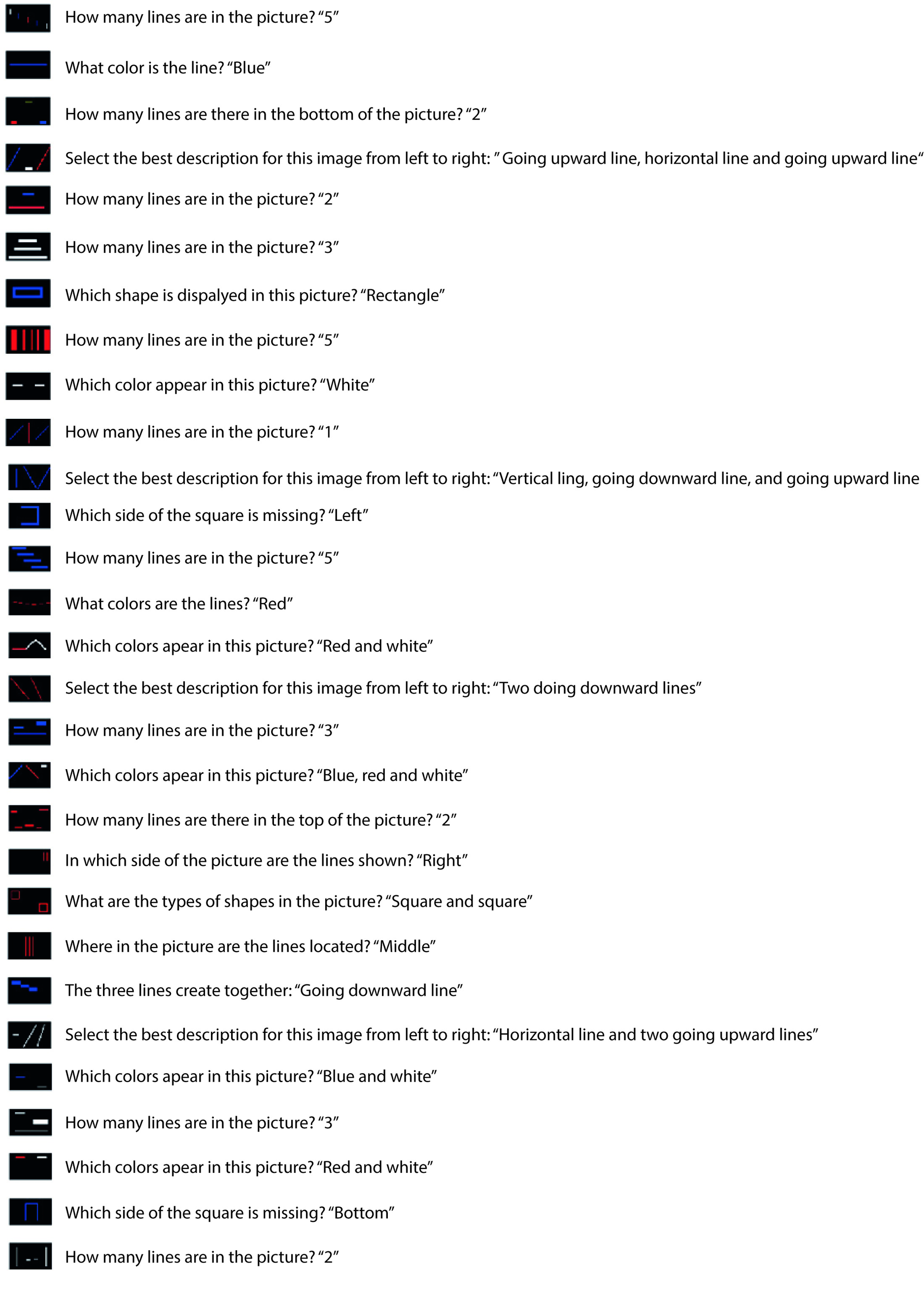

Supplement: S2 Fig — (TIF) [file pone.0250281.s002.tif]
